# The Impacts of Engagement in the Latino Medical Student Association

**DOI:** 10.1089/heq.2022.0099

**Published:** 2023-02-16

**Authors:** Sunny Nakae, Stacey Martinez, Jordan J. Juarez, Cintya Beltran Sanchez

**Affiliations:** ^1^ Department of Medical Education, California University of Science and Medicine, Colton, California, USA.; ^2^ Swedish Cherry Hill Family Medicine Residency Program, Seattle, Washington, USA.; ^3^ Lewis Katz School of Medicine at Temple University, Philadelphia, Pennsylvania, USA.; ^4^ University of California Riverside School of Medicine, Riverside, California, USA.

**Keywords:** LMSA, medical education, medical school cocurricular groups, Latinx, student career support, institutional support for Latinx students, LHS+

## Abstract

**Importance:**

The Latino Medical Student Association (LMSA) is a student-run national organization founded in 1972 dedicated to recruiting and retaining members enrolled in health professions programs through academic and social support activities. This study investigates the career impact of member participation in LMSA.

**Objective:**

To determine if engagement in LMSA at the individual and school levels contributes to retention, success, and commitment to underserved communities.

**Design:**

A voluntary online retrospective 18-question survey sent to LMSA-member medical students in the United States and Puerto Rico from the graduating classes of 2016–2021.

**Setting:**

Students in medical schools in the United States and Puerto Rico.

**Main Outcomes and Measures:**

There were 18 survey questions. A total of 112 anonymous responses were collected from March 2021 to September 2021. The survey queried levels of engagement with the LMSA and agreement on questions related to support, belonging, and career development.

**Results:**

There is a positive relationship between level of engagement in the LMSA and social belonging, peer support, career networking, community engagement, and career commitment to serve Latinx communities. These positive outcomes were enhanced for respondents reporting strong support for their respective school-based LMSA chapters. We did not find a significant relationship between participation in the LMSA and research experiences during medical school.

**Conclusions and Relevance:**

Participation in the LMSA is associated with positive individual support and career outcomes for members. Supporting the LMSA as a national organization and within school-based chapters can increase support for Latinx trainees and enhance career outcomes.

## Introduction

The Latino Medical Student Association (LMSA) is a 501 (c)(3) nonprofit organization founded to represent, support, educate, and unify medical trainees, especially those who identify as Latino/a, Hispanic, Spanish origin, and/or additional intersecting identities (LHS+).^1^ The LMSA's mission is to unite and empower current and future physicians through service, mentorship, and education to advocate for the improved health of the LHS+ community in the United States. The LMSA has >150 medical school chapters and 171 academic advisors across five regions (Northeast, West, Midwest, Southeast, and Southwest) encompassing all 50 states. LHS+ students (identifying as LHS+ ethnicity alone or in combination with other races and/or ethnicities) comprised 11.6% of the applicant pool to allopathic medical schools in 2021–2022.^2^

Among matriculants,^3^ LHS+ students represent 12.6% (6.9% reporting LHS+ ethnicity alone and 5.7% reporting LHS+ ethnicity in combination with other ethnicities and/or races). In 2021, LHS+ students (alone or in combination) were 10.4% of all graduates.^4^ The small percentage of attrition over 4 years (2.2%) equals ∼498 LHS+ trainees that may not enter the physician workforce despite meeting the rigorous preparation standards required for admission.

The distribution of LHS+ students across medical schools also indicates that many schools lack a critical mass, and that LHS+ matriculants may be experiencing high levels of stereotype and belongingness threat based on class compositions. According to the 2020 Medical School Admissions Requirements Online, 23 of 154 schools had <4% of their entering classes identify as all or part LHS+, and 46 schools had <6% identify as all or part LHS+.^5^ Just 28 schools (18%) had >17% of their entering classes identify as all or part LHS+. The total enrollment of LHS+ students at the four schools in Puerto Rico is equal to the total LHS+ enrollment of the next *62 schools*. The uneven distribution of students across schools may create social belonging and support challenges for LHS+ students that students from highly represented and historically included groups may not experience.

Historically excluded students, including LHS+ students, are more likely to leave medical school before graduation.^6^ Although many of these withdrawals can be attributed to academic reasons, the gaps in social, emotional, and structural support for historically excluded trainees can be more pronounced for students likely to have intersectional vulnerabilities, such as first-generation status and low-income families of origin.^7–10^ Although the final reason may be underperformance, the structural reasons contributing to it may not be captured in withdrawal data.

For many students, there is a gap in vital support and resources once enrolled. Underrepresented students reported experiencing less social support and reported having less positive learning environments.^11^ Underrepresented students make up nearly half of all medical students who failed the United States Medical Licensing Examination (USMLE) Step 1 on the first try, which indicates increased need for support.^12^ Being underrepresented in a medical school class places a student at increased risk for academic difficulty, including delayed graduation, license examination failure, and course failure due to structural, social, and emotional inadequacies of support.

The LMSA as a national organization has played a vital role in creating a professional home and identity for LHS+ trainees. For students participating in their local chapters, the LMSA can provide social connection, emotional support, opportunities to be mentored and to mentor others, and a platform to engage in meaningful cultural and community endeavors, all within a safe environment that is not reproducible in other medical school settings. Underrepresented minority students are more likely to partake in research and mentorship activities that align with their identities and improve their communities.^13,14^

LHS+ medical students are uniquely positioned not only to deliver culturally appropriate care to a growing LHS+ population, but also to enrich a medical school student body and serve as advocates for LHS+ health issues. Underrepresented minority students are more likely than their White peers to work in the community in which they identify, and typically in underserved areas.^15^ Role models, career support, and experiencing social well-being are critical for success in medicine, all of which can be obtained through LMSA through LHS+ focused initiatives and opportunities that are not universally offered by medical school curriculums across the country.

Social Cognitive Career Theory (SCCT) forms the framework for this exploratory study of outcomes related to engagement in the LMSA. SCCT asserts that career development takes place within the context of exposure and interest, career choice, performance, and persistence.^16^ LHS+ students may face barriers to developing career interest as premedical students, as they may lack role models and visible examples. SCCT posits that seeking a career is undergirded by exposure to that career in the first place. During medical school, LHS+ students may face additional barriers in specialty choice due to sparse representation and presence of LHS+ faculty. Messages that students receive about their potential success, as transmitted in peer and identity role models, may shape student career choices and perceptions of their probable success, and by extension career choices.

Overall performance and persistence for LHS+ students may be influenced by contextual factors such as stereotype threat that may arise in settings where belonging is threatened simply by being few in number.^17^ Organizations such as the LMSA may serve to promote greater career success by providing peer and faculty support and role models, creating a community to foster a sense of belonging, and facilitating career exploration and exposure through trusted networks. Given the historical exclusion and underrepresentation of LHS+ individuals in academic medicine, understanding factors that promote participation and career advancement is critical. Leaders in academic medicine have long called for support and change to further diversify medicine with stronger representation from LHS+ communities. This study sought to understand the extent to which the LMSA may be an effective organizational tool for the advancement of LHS+ trainee careers.

## Overview

This national study was a voluntary online survey created to assess the impact of LMSA on medical student careers. There were 112 respondents, including LMSA-member medical student graduates from the classes of 2016 through 2021 and current 4th year LMSA-member medical students. The responses were anonymous and participants had the option to withdraw from the study at any time. The estimated time of completion was 5 min. The study was conducted from March 2021 to September 2021 and was approved as exempt by the Institutional Review Board at the California University of Science and Medicine (Protocol HS-2021-04).

## Questions

There were a total of 18 questions, including multiple choice options, check-box options, and 5-point ordinal response options indicating levels of agreement with statements. In addition, there were three open text questions where participants could directly type answers.

## Survey Administration

A study flier and clickable link were shared on multiple social media platforms to recruit participants, including the National LMSA Instagram and Twitter pages, and through personal author Instagram and Twitter pages. In addition, the study flier and clickable link were sent through e-mail from the National Hispanic Medical Association to current residents and to the list of national advisers for the LMSA chapters to share with their networks. Individual e-mails were sent to potential participants from the authors as well.

## Results

The only demographic data obtained were the medical school graduation year and first in family data. No names, medical school institutions, or other identifiable data were obtained. Participants were recruited from medical schools and residencies across the country. Of the 112 participants, 23% were from the Class of 2022, 37% were from the Class of 2021, 11% were from the Class of 2020, 6% were from the Class of 2019, 5% were from the Class of 2018, 4% were from the Class of 2017, 5% were from the Class of 2016, and 9% were LMSA members who would be graduating after 2021. Ninety-one percent of those that completed the survey were the first in their family to go to medical school.

In terms of when participants became aware of LMSA, 35% became aware as premedical LMSA+ students, 55% became aware as 1st-year medical students, 5% became aware as 2nd-year medical students, and 5% became aware as 3rd-year medical students. Sixty-nine percent of the participants regularly participated in the LMSA, 15% participated fairly regularly, 13% participated rarely, and 3% did not participate. Nearly all respondents (99%) affirmed their intention to serve LHS+ populations in their medical careers. Finally, slightly more than three quarters of the respondents (76%) indicated their intentions to remain involved with the LMSA in residency and post-residency.

Quantitative analysis using *t*-tests to compare students with lower participation versus those with higher participation in the LMSA found that the level of participation significantly impacted outcomes (Table 1). Respondents reported community and social support areas higher when participation was regular or fairly regular except for questions 14 and 15, which were related to enhanced career opportunities and/or specialty exploration and scholarly activities and/or research opportunities, respectively. LMSA members who reported a high level of institutional support for their engagement in the LMSA in comparison with those who reported all other support levels reported more favorable impacts in all areas except scholarly activities and research opportunities (Table 2).

**Table 1.** Survey Outcomes by Participation Level

Questions	Did not participate	Rarely participated	Participated fairly regularly	Participated regularly	*p*
*N*	3	15	17	77	
7. The LMSA gave me the opportunity to experience community connection during medical school.	3.67 (1.15)	3.00 (1.41)	4.41 (0.87)	4.75 (0.52)	<0.001
8. The LMSA provided social support with peers during medical school.	3.67 (1.15)	3.27 (1.16)	4.35 (0.93)	4.66 (0.64)	<0.001
9. The LMSA gave me the opportunity to receive emotional support from peers during medical school.	3.00 (0.00)	3.20 (1.32)	4.12 (1.05)	4.58 (0.71)	<0.001
10. The LMSA helped me identify mentors and role models during medical school.	2.67 (1.53)	3.00 (1.36)	3.29 (1.45)	4.12 (1.01)	<0.001
11. The LMSA provided PEER networking opportunities during medical school.	3.00 (0.00)	3.73 (1.28)	3.76 (1.44)	4.53 (0.72)	<0.001
12. The LMSA provided FACULTY networking opportunities during medical school.	3.33 (0.58)	2.60 (1.24)	3.41 (1.42)	3.71 (1.11)	0.011
13. The LMSA provided enhanced learning opportunities during medical school.	2.67 (0.58)	3.20 (1.52)	3.24 (1.68)	4.13 (0.92)	0.001
14. The LMSA provided enhanced career opportunities and/or specialty exploration opportunities during medical school.	2.67 (0.58)	3.13 (1.41)	3.47 (1.28)	3.73 (1.14)	0.166
15. The LMSA gave me the opportunity to engage in scholarly activities and/or research during medical school.	2.67 (0.58)	3.20 (1.08)	3.29 (1.16)	3.56 (1.07)	0.323
16. The LMSA strengthened my ability to improve the health of underserved communities.	3.00 (0.00)	3.47 (1.25)	4.00 (1.06)	4.62 (0.69)	<0.001

*p*-Values for did not and rarely compared with fairly regularly and regularly.

LMSA, Latino Medical Student Association.

**Table 2.** Level of Active Institutional Support for the Latino Medical Student Association and Outcomes

Questions	High support, mean (SD)	All other support, mean (SD)	*p*
*n*	58	54	
7. Community connection	4.76 (0.57)	4.09 (1.17)	<0.001
8. Peer social support	4.76 (0.54)	4.02 (1.07)	<0.001
9. Peer emotional support	4.57 (0.73)	3.98 (1.16)	0.002
10. Identify mentors and role models	4.16 (0.91)	3.43 (1.41)	0.001
11. Peer networking	4.62 (0.64)	3.89 (1.19)	<0.001
12. Faculty networking	3.95 (0.96)	3.04 (1.29)	<0.001
13. Enhanced learning	4.19 (1.00)	3.44 (1.33)	0.001
14. Enhanced career and/or specialty exploration	3.81 (1.07)	3.33 (1.30)	0.036
15. Enhanced scholarly activities and/or research	3.53 (1.05)	3.35 (1.12)	0.374
16. Improve the health of underserved communities	4.60 (0.70)	4.04 (1.10)	0.001

SD, standard deviation.

Given these results, it is clear the LMSA plays a vital part in the lives of those involved in the organization during their time in medical school. In addition, when the sample was divided into those that found out about the LMSA as premedical students, compared with those who joined in medical school, no difference was found in the outcomes. Whether members joined the LMSA as a premedical student or as a medical student, the community and social support benefits reported were statistically indistinguishable.

Participants shared their personal experiences with the LMSA, the strengths of the organization, and areas the LMSA can improve in the free text space provided. Participants' responses highlight both the manifest and latent functions of the organization. Common themes among participants include peer camaraderie, various support systems otherwise not offered by the participants' home institutions, and leadership development.

- “LMSA was my family during medical school. I leaned so many times on colleagues I met through this organization rather than my home institution.”
- “[The LMSA] helped me build confidence in myself by seeing other Latinas in leadership.”
- “I loved being a part of LMSA throughout my medical school experience. Not only did it give me leadership opportunities, but it also strengthened my desire to serve the Latinx patient population while helping me build the skills to be able to do so.”

Overall, the LMSA positively impacted the participants' medical school experiences and enhanced their personal development as physicians in training. The LMSA offered networking opportunities, academic support when students struggled, social support for those experiencing isolation, and a safe space where participants could relate to one another's struggles as underrepresented in medicine.

- “As a premed, [LMSA] shaped my idea of a physician and medical student. [In medical school] When I struggled academically, LMSA mentors were a trusted source of information and support that were key to my success.”
- “The LMSA was there for me when I needed someone to tell me ‘it's going to be okay, you can do this.’ As an M1, the support of the M2-M4s, as well as watching those ahead of me ‘make it,’ was the strength and inspiration I needed to know I could do it too when my medical school trajectory wasn't going as planned. Additionally, though I knew medicine to be an institution with deep roots in racism, I was unprepared for the racism I would see daily. Having a community who I knew was strong and facing the same challenges, but persevering and making changes, was key.”

Participants also commented on areas for the LMSA to improve, including greater institutionalized support, and more staff, students, and faculty of LHS+ backgrounds. There was a strong sentiment to increase collaboration with larger organizations as a means of advocacy and policy work, improving retention after undergraduate medical education, and increasing engagement among alumni, faculty, attending physicians, and residents. LMSA members commented on the benefits that LHS+ students brought to their institutions, such as Spanish language skills, recruitment endeavors, developing curriculum to increase skills for culturally appropriate care, serving LHS+ patients and communities, and volunteering as mentors.

- “LMSA helps us open our eyes to issues that our schools may not teach us about; develop as leaders; find strength in numbers to support causes that we are passionate about; find social and emotional support; and become advocates for health equity.”
- “LMSA provided the education we simply don't receive in the classroom (or at least we should be, but certainly not via the ‘diversity’ education).”

## Limitations

Limitations of the study include the relatively small response rate and the sampling bias among active LMSA members. The COVID-19 pandemic's strain on graduate medical education trainees in the health care system may have played a role in the small response rate among residents. In addition, LMSA members who saw the organization as beneficial may have been more motivated to complete the survey. There were no incentives offered for survey completion, which may have further skewed the sample toward those who felt positively toward the LMSA. The lack of LMSA alumni organizational structure made contacting current graduate medical education trainees and early career faculty difficult; therefore, the sample size of graduates from more distant years was quite small within the sample.

## Discussion and Implications

Findings of the survey of LMSA members indicate positive outcomes for participation in and support of the LMSA. Given the shortage of providers in medicine, every applicant and matriculant should receive the resources and support necessary to achieve success. Career development and mentorship through LMSA networking was shown to be effective for members who engaged, therefore facilitating more interaction for LMSA members with residents and early career faculty may also be a promising practice for retention and encouragement of career goals.

Furthermore, the lack of significant findings in the survey regarding participating in the LMSA and identifying specialty exploration opportunities highlights the sparse representation of LHS+ faculty members within medicine. Institutions should continue to invest in faculty development and programs that encourage LHS+ trainees to pursue academic medicine. The nonsignificant finding for scholarly activities and research opportunities also highlights the need for greater support for LHS+ faculty and programs that encourage early engagement in academic medicine and scholarship.

Given the lack of significant findings with regard to engagement with LMSA and specialty exploration, coupled with nonsignificant findings of engagement with LMSA and ability to find scholarly and research opportunities, the organization should seek guidance from other minority medical student organizations with similar missions, such as the Student National Medical Association (SNMA). Founded in 1964 as a subdivision of the National Medical Association, the SNMA similarly supports underrepresented minority medical students, particularly Black medical students, through mentorship initiatives and conferences across 196 local chapters at medical schools across the country.^18^

Other findings of this study showed that 91% of survey respondents reported being the first in their family to attend medical school, which also frames the LMSA as an important support mechanism and community for students from first-generation and low-income families of origin in medical education. Given the large percentage of respondents who were first-generation medical students, the results may be applicable to support systems for students who do not come from families with generations of physicians. Another critical finding of this study was the fact that the overwhelming majority of respondents indicated an intention to serve LHS+ patient populations in their medical careers, consistent with previously published literature indicating that underrepresented minority students are more likely to work in underserved communities compared with their White peers.^15^

Future studies could explore various medical student outcomes for LHS+ students who engage with LMSA during the 4 years of medical school compared with those who do not, such as academic outcomes. Assessing residency placement, USMLE Steps 1 and 2 pass rates, or percentage of students pursuing careers in academic medicine among LMSA members compared with nonmembers could provide data for areas of improvement for the organization. Other potential studies could assess supportive outcomes for members who participated in LMSA for all 4 years compared with those who did not, such as perceived emotional support or frequency of anxiety or depressive symptoms.

More scholarship is needed to understand the experiences of LHS+ students. The LMSA member list serve could be an avenue for that, similar to how the SNMA used their list serve to examine microaggressions among minority medical students, as well as to assess the relationship between experiences of discrimination, institutional responses to seminal race events, and depressive symptoms among Black medical students.^19–21^ The results from similar studies on LHS+ trainees could provide medical schools with data on the utility of LMSA, as well as opportunities for organizational growth.

The mission, focus, and activities of the LMSA clearly align with the goals of many institutions to train a physician workforce committed to caring for the underserved and addressing health disparities. The LMSA provides support for LHS+ trainees, a critical function. It also strengthens and affirms the commitment of those trainees to serve LHS+ communities. Based on these findings, institutions can increase social, emotional, and career support for LHS+ trainees by supporting the LMSA, both nationally and within their local institutions.

## Abbreviations Used

LHS+: Latino/a, Hispanic, Spanish origin, and/or additional intersecting identities
LMSA: Latino Medical Student Association
SCCT: Social Cognitive Career Theory
SD: standard deviation
SNMA: Student National Medical Association
USMLE: United States Medical Licensing Examination
